# Cobra Three-Finger Toxins Interact with RNA and DNA: Nucleic Acids as Their Putative Biological Targets

**DOI:** 10.3390/ijms26094291

**Published:** 2025-05-01

**Authors:** Alexey V. Osipov, Vladislav G. Starkov, Victor I. Tsetlin, Yuri N. Utkin

**Affiliations:** Shemyakin-Ovchinnikov Institute of Bioorganic Chemistry of the Russian Academy of Sciences, Moscow 117997, Russia; osipov-av@ya.ru (A.V.O.); vladislavstarkov@mail.ru (V.G.S.); victortsetlin3f@gmail.com (V.I.T.)

**Keywords:** three-finger toxin, nucleic acid, electrophoretic mobility shift assay, α-neurotoxin, cytotoxin, non-conventional toxin, RNA, DNA

## Abstract

Three-finger toxins (TFTs), including neurotoxins and cytotoxins, form one of the largest families of snake venom proteins and interact with various biological targets. Neurotoxins target proteinaceous receptors while cytotoxins interact mainly with the lipids of cell membranes and to a lesser extent with carbohydrates. However, no data about the interaction of TFTs with nucleic acids can be found. To detect this interaction, we applied spectrophotometry, ion-paired HPLC and electrophoretic mobility shift assay (EMSA). Using spectrophotometry, we found that TFTs from cobra venom increased the optical density of an RNA solution in a time-dependent manner indicating toxin interaction with RNA. A decrease in the net negative charge of the RNA molecule upon interaction with neurotoxin II from cobra venom was revealed by ion-pair HPLC. EMSA showed decreased electrophoretic mobility of both RNA and DNA upon addition of different TFTs including the non-conventional cobra toxin WTX and water-soluble recombinant human three-finger protein lynx1. We suggest that the interaction with nucleic acids may be a common property of TFTs, and some biological effects of TFTs, for example, cytotoxin-induced apoptosis in cancer cell lines, may be mediated by interaction with nucleic acids.

## 1. Introduction

Cobra venom is a complex mixture of proteins comprising both enzymes and enzymatically inactive compounds. Proteomic data have shown that the latter are predominantly represented by the three-finger toxin (TFT) family—α-neurotoxins of “long-” and “short-chain” types, cytotoxins and some other less abundant toxins [1,2,3]. Cobra TFTs are small proteins of less than 9 kDa that have a high similarity in the secondary and tertiary structure. The conservative structure provides them with high resistance to proteolysis and physical effects but does not impede the display of hugely different biological activities against a wide variety of targets in the body of victims [4]. The main targets of α-neurotoxins are ligand-gated ion channels, mainly nicotinic acetylcholine receptors, which are transmembrane multi-subunit glycoproteins with a molecular mass over 100 kDa [5,6]. Some of the biological activities of TFTs cannot be explained by binding with their main “classic” target only. In particular, “short-chain” α-neurotoxins block the nicotine acetylcholine receptors of the muscle, but their analgesic properties are interpreted as a targeted effect on the muscarinic system of the brain [7]. Most of the evidence accumulated to date indicates that the main target of cytotoxins is the cell membrane. In particular, the lipids of cell membranes are the primary target [8,9]. However, it should be mentioned that the interaction of cytotoxins with carbohydrates has been also demonstrated [10,11]. Cytotoxins can also exert an analgesic effect, which is due to inhibiting the phosphorylation of intracellular proteins but not as a direct result of cytolysis [12]. Interestingly, cytotoxins may interact with nucleotides [13] and oligonucleotides [14], but no data about the interaction of TFTs with nucleic acids can be found.

However, the toxins, which affect nucleic acids, are present in cobra venoms and include mostly nucleic acid-degrading enzymes. These are nucleases (ribonuclease and phosphodiesterase) [15,16], nucleotidases (5′-nucleotidase and ATPase) [17] and phosphomonoesterases [18].

Among these enzymes, ribonucleases (RNases) are practically not investigated, although their presence in cobra venom has long been described [19]. Therefore, their structure is still completely unknown, and this may be the reason why they have not been detected in the proteomic research on cobra venoms. In our previous research on snake toxins, we attempted to isolate and characterize RNase from cobra venom [20]. To measure RNase activity, we used a spectrophotometric method, based on the registration of an increase in the optical density of an RNA solution under the reaction with RNase [21,22,23]. After the separation of cobra venom by gel filtration, we observed that along with the putative RNase activity detected by this method in fractions corresponding to proteins with molecular masses of 12–15 kDa and more than 30 kDa, the highest increase in the optical density was detected in fractions containing proteins with molecular masses of less than 9 kDa [20]. It seemed questionable that the latter are RNases, given the fact that known RNases have a molecular mass over 11 kDa [24]. In cobra venoms, the most abundant proteins with a molecular weight below 9 kDa are TFTs. We hypothesize that TFTs, by interacting with RNA, can somehow mimic RNase activity when determined by spectrophotometric methods.

The aim of this work is to find out whether TFTs from cobra venom can interact with nucleic acids—natural RNA and DNA. Here, we report a time-dependent increase in the optical density of an RNA solution after the addition of certain TFTs, i.e., neurotoxins and cytotoxins from the venom of the Middle Asian cobra *Naja oxiana*; a change in the RNA chromatographic profile after incubation with a cobra neurotoxin; and a slowdown in the electrophoretic mobility of the RNA and DNA in the presence of TFTs, detected by agarose gel electrophoretic mobility shift analysis (EMSA). The data obtained suggested that TFTs could interact with RNA and DNA. It should be mentioned that many new properties, sometimes unexpected, have been discovered for snake TFTs recently [25]. In this report, we expand the range of activities inherent in TFTs and for the first time show their interaction with natural nucleic acids.

## 2. Results

### 2.1. Spectrophotometry Measurements

**Figure 1:**
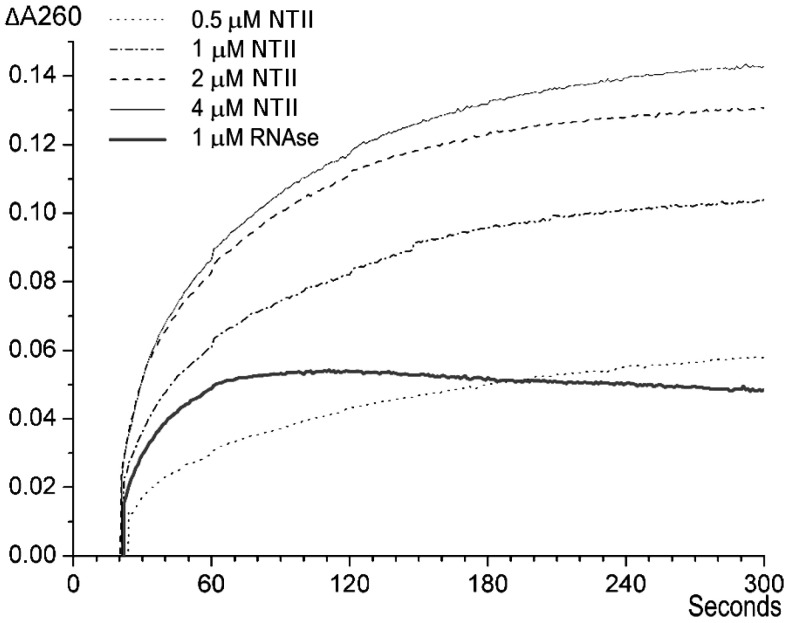
Time-dependent changes in the optical density at 260 nm (ΔA260) of a yeast RNA solution (1 optical density unit) after the addition of a 1% solution of neurotoxin II (NT II) to a final concentration of 0.5, 1, 2 and 4 μM or bovine pancreatic RNase A to a final concentration of 1 μM (bold line). The background adsorption was subtracted at each curve registration. In this and the following figures, the results of one of at least three independent experiments are presented.

In an attempt to isolate RNase, we analyzed cobra venom. We first used the method based on the increase in optical density upon RNase digestion of nucleic acid [21,22,23]. The data obtained by us earlier showed that the most abundant venom component(s) producing an increase in the optical density of an RNA solution, which suggests RNase activity, possess(es) molecular mass in the range of 6–8 kDa [20]. In view of these data, we hypothesized that these may be TFTs, which have molecular masses exactly in this range and are quite abundant in cobra venom. To test this hypothesis, we chose several TFTs from the venom of cobra *N. oxiana*, which included “long-chain” neurotoxin I (NTI) with a molecular mass of 8.0 kDa, “short-chain” neurotoxin II (NTII) of 6.9 kDa, cytotoxin I (CTI) of 6.8 kDa and cytotoxin II (CTII) of 6.6 kDa, as well as non-conventional toxin (WTX) of 7.6 kDa from the venom of cobra *N. kaouthia*. Each toxin was added to a yeast RNA solution, and changes in optical density at 260 nmwere registered. In Figure 1, we provide the data obtained for NTII as an example. After being added to the diluted solution of the yeast RNA, the neurotoxin increases the solution optical density over 3–5 min to a level that depends on the toxin concentration. The optical density does not change over the next 5–6 min and then begins to decrease very slowly, probably due to the precipitation of the reaction mixture components. Similar changes, but several times faster, occur when the RNA solution is mixed with a bovine pancreatic RNase A solution (Figure 1, bold line).

**Figure 2:**
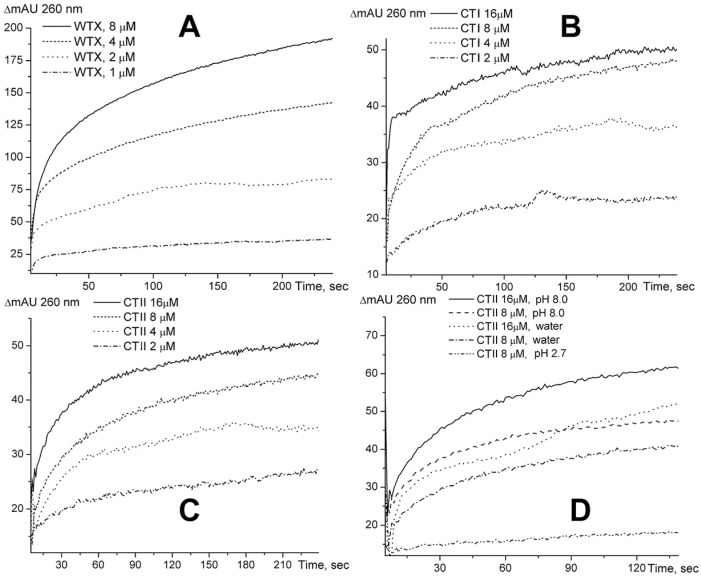
Time-dependent changes in the optical density at 260 nm (ΔmA260) of a yeast RNA solution (1 optical density unit) after the addition of a 1% solution of WTX (**A**), CTI (**B**) and CTII (**C**) to the final concentrations indicated at each panel. The dependence of the changes in the optical density on pH for CTII (**D**). The background adsorption was subtracted at each curve registration.

We also investigated the effects of other TFTs mentioned above on yeast RNA using a spectrophotometric method and found that WTX increases the optical density of the RNA solution in about the same way as NTII (Figure 2A), while CTI and CTII exert weaker effects (Figure 2A and Figure 2B, respectively).

Interestingly, the changes in the optical density depend on the pH of the reaction mixture. The optical density increases more at alkaline pH values (Figure 2D). This may be explained by the stronger ionization of the nucleic acid in the alkaline solution, which apparently leads to stronger binding of positively charged toxin molecules.

**Figure 3:**
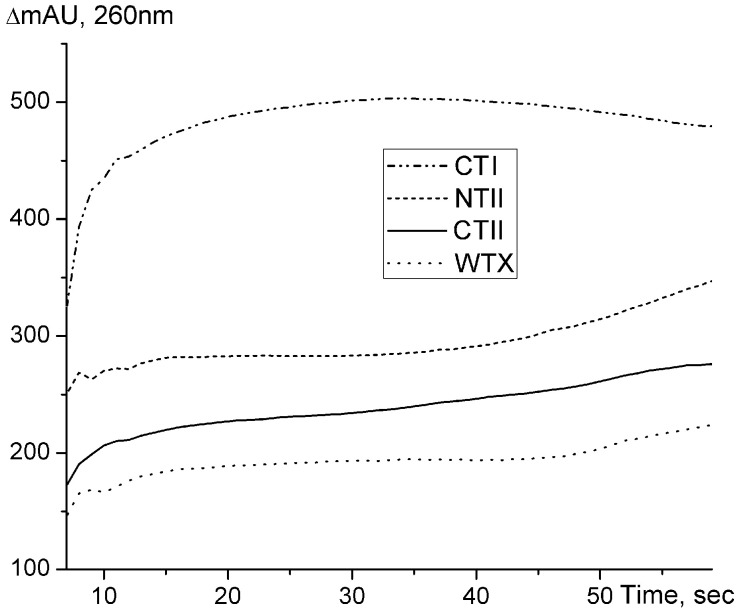
Time-dependent changes in the optical density at 260 nm (ΔmA260) of a solution of high-molecular DNA from cattle spleen (1 optical density unit) after the addition of a 1% solution of toxins to a final concentration of 8 μM. The background adsorption was subtracted at each curve registration.

The increase in optical density at 260 nm was also observed when the toxins were added to high-molecular DNA from cattle spleen (Figure 3). However, in the case of DNA, the greatest increase in optical density was observed upon the addition of CTI, which probably reflects differences in the structure of the resulting complexes.

### 2.2. Ion-Paired Reversed-Phase HPLC

**Figure 4:**
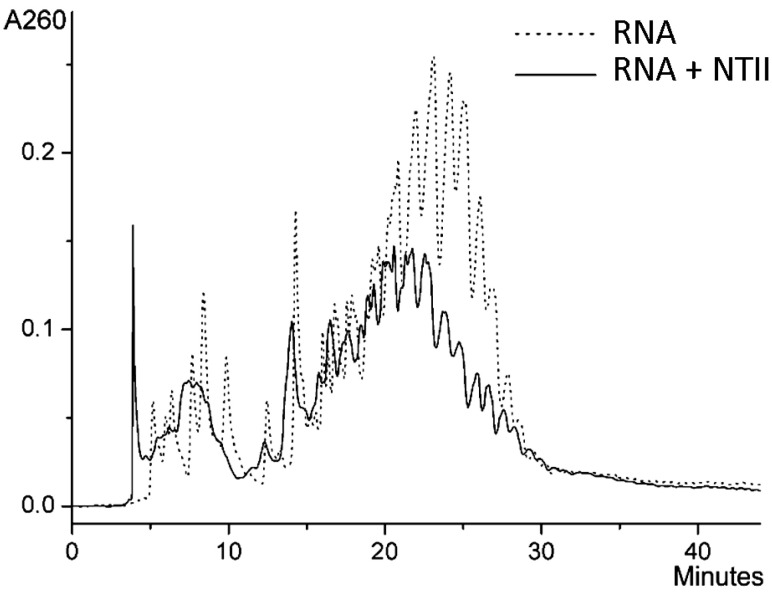
Separation of the products of NTII interaction with yeast RNA by ion-paired reversed-phase HPLC on a Phenomenex C18 Jupiter (4.6 × 250 mm) column (solid line). An acetonitrile gradient from 2 to 22% in a 0.1 M triethylammonium acetate buffer (pH 7.0, 0.1% Tween 20) for 40 min was used. The profile for RNA after incubation with 0.1% Tween 20 without NTII is shown as a dotted line.

To determine further whether the neurotoxin interacts with RNA, we used ion-pair reverse-phase HPLC [26]. It was shown earlier that when a gradient of acetonitrile in triethylammonium acetate buffer is used in this chromatography, nucleic acids elute from the column generally in order of increasing chain length [27,28]. Since the products of the interaction of neurotoxin with RNA partially precipitated during the incubation of the reaction mixture for several hours, 0.1% Tween 20 was added to prevent precipitation. Before chromatography, we verified that the detergent at this concentration did not affect the results of spectrophotometric measurements used to record the interaction of RNA with the neurotoxin (Figure S1). As expected, analysis of yeast RNA by ion-paired reversed-phase HPLC showed a high degree of heterogeneity (Figure 4, dotted line). This is not surprising, as the RNA used contains chains of different length. In further experiments, RNA was incubated overnight with NTII in the presence of a detergent. After incubation with the neurotoxin, the chromatographic profile changed strongly. A shift in the chromatographic profile toward lower eluent concentrations is evident (Figure 4). The set of maximum intensity peaks at 21–27 min is shifted to the region of 18–25 min. This may be explained by the weaker interaction of the RNA–neurotoxin complex with positively charged ion-pair reagent triethylammonium because of the decrease in the net negative charge of the complex as compared to RNA alone. Thus, chromatographic data evidence in favor of the interaction of NTII with RNA.

### 2.3. Electrophoretic Mobility Shift Assay

**Figure 5:**
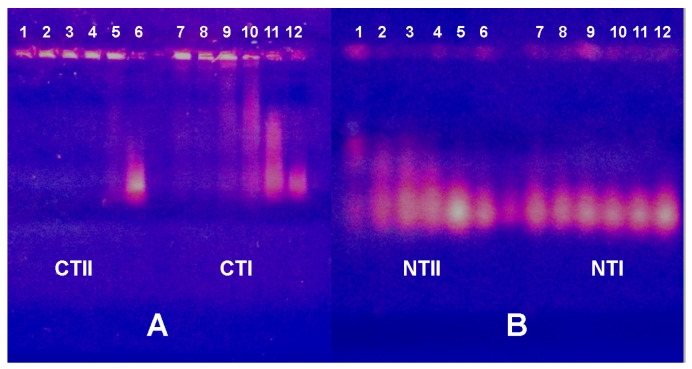
Electrophoretic mobility shift assay (EMSA) of the TFTs influence on electrophoretic motility of DNA. In (**A**), cytotoxins II and I (CTII and CTI) are added in lanes 1–5 and 7–11, respectively. In (**B**), neurotoxins II and I (NTII and NTI) are added in lanes 1–5 and 7–11, respectively. Control samples (10 μL 0.1% DNA) are in lanes 6 and 12. The nucleic acid samples were pre-incubated with 4 (lanes 1 and 7), 2 (lanes 2 and 8), 1 (lanes 3 and 9), 0.5 (lanes 4 and 10) and 0.125 (lanes 5 and 11) μL of a 0.5% solution of the respective toxin. In this and the following figures, the contrast was increased for better visibility. The black and white versions of this and the following figures are included in the Figures S2–S7.

**Figure 6:**
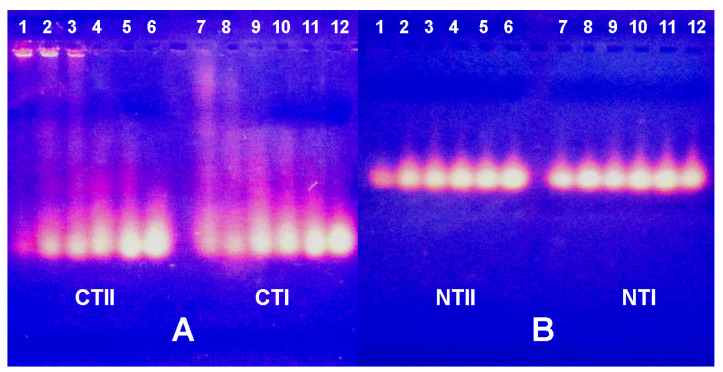
Electrophoretic mobility shift assay (EMSA) of the TFTs influence on electrophoretic motility of RNA. In (**A**), cytotoxins II and I (CTII and CTI) are added in lanes 1–5 and 7–11, respectively. In (**B**), neurotoxins II and I (NTII and NTI) are added in lanes 1–5 and 7–11, respectively. Control samples (6 μL 0.6% RNA) are in lanes 6 and 12. The nucleic acid samples were pre-incubated with 4 (lanes 1 and 7), 2 (lanes 2 and 8), 1 (lanes 3 and 9), 0.5 (lanes 4 and 10) and 0.125 (lanes 5 and 11) μL of a 0.5% solution of the respective toxin.

Among various methods for the investigation of nucleic acid–protein interaction, the electrophoretic mobility shift assay (EMSA, also known as the gel mobility shift or gel retardation assay) is widely used and considered a simple and very efficient method [29,30]. We used this approach to investigate changes in the electrophoretic motility of both yeast RNA and cattle spleen DNA in agarose gel after incubation with NTI, NTII, CTI and CTII (Figure 5 and Figure 6).

Among the toxins tested, CTII manifested the highest affinity to DNA; at all concentrations used, it bound DNA so strongly that the complexes formed did not leave the gel pockets (Figure 5A, lanes 1–5). CTI formed less stable complexes with DNA, as it suppressed DNA migration only at high concentrations (Figure 5A, lanes 7, 8). At lower CTI concentrations, the CTI-DNA complexes of different stoichiometry were seen (Figure 5A, lanes 9–11). Such complexes were also well seen in the case of NTII (Figure 5B, lanes 1–5), while NTI did not display any visible effect (Figure 5B, lanes 7–11).

Similar electrophoretic patterns were observed with RNA but at higher concentrations of toxins (Figure 6). So, DNA mobility in the presence of TFTs was reduced more than that of RNA. This suggests that DNA forms a more stable complex with toxins than RNA.

**Figure 7:**
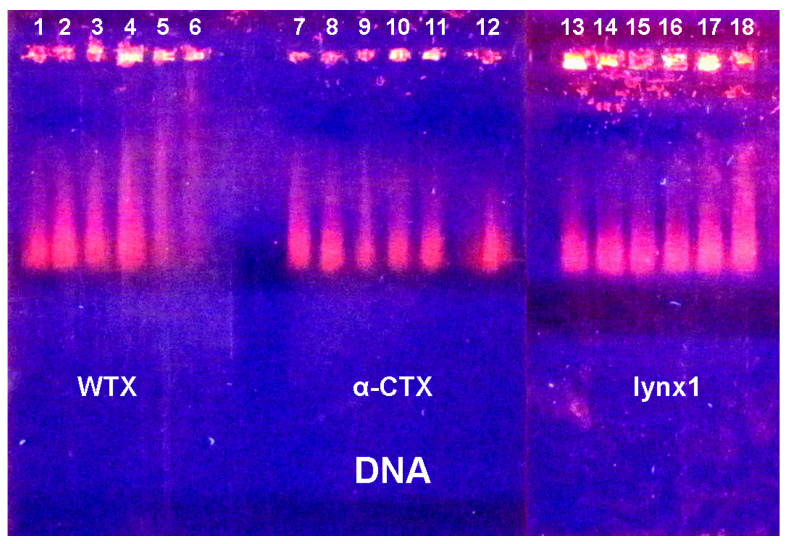
Electrophoretic mobility shift assay (EMSA) of the WTX (**left**), α-CTX (**center**) and lynx1 (**right**) influence on the electrophoretic motility of DNA. Lanes 1, 12 and 13—control samples of 10 µL DNA (1 mg/mL); lanes 2, 11 and 14—DNA with 0. 25 µL, lanes 3, 10 and 15—with 0.5 µL, lanes 4, 9 and 16—with 1 µL, lanes 5, 8 and 17—with 2 µL and lanes 6, 7 and 18—with 4 µL of 0.5% WTX, α-CTX or lynx1, respectively.

**Figure 8:**
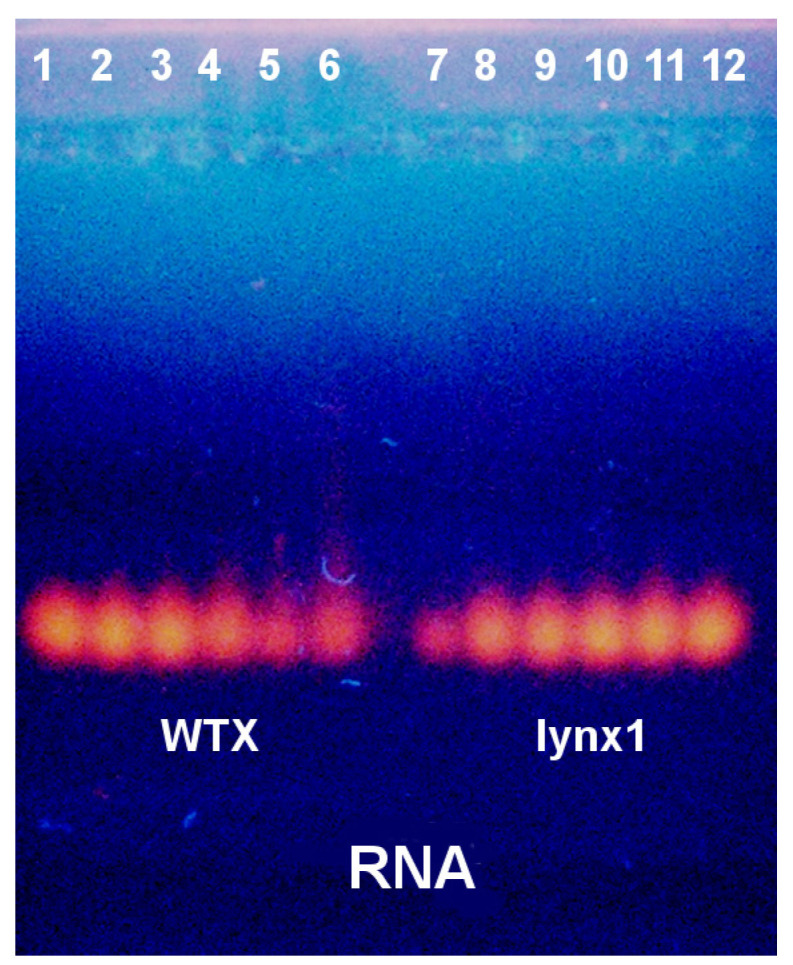
Electrophoretic mobility shift assay (EMSA) of the WTX (**left**) and lynx1 (**right**) influence on the electrophoretic motility of RNA. Lanes 1 and 12—control samples of 6 µL RNA (6 mg/mL); lanes 2 and 11—RNA with 0. 25 µL, lanes 3 and 10—with 0.5 µL, lanes 4 and 9—with 1 µL, lanes 5 and 8—with 2 µL and lanes 6–7—with 4 µL of 0.5% WTX or lynx1, respectively.

Two more TFTs including “long-chain” α-cobratoxin (α-CTX) and non-conventional toxin WTX from cobra *N. kaouthia* venom were tested for their capacity to interact with DNA. A water-soluble analog of endogenous protein lynx1 possessing a three-finger fold was also tested for its capacity to interact with DNA and RNA. Using EMSA, we found that all three proteins formed complexes with nucleic acids (Figure 7 and Figure 8). It is evident that all proteins slow down DNA movement. Of the three proteins, DNA movement is slowed most strongly by WTX, followed by α-CTX, with lynx1 being the least active (Figure 7). In this case, lynx1 has an effect only at the highest concentration. Thus, WTX binds DNA most strongly and lynx1 least strongly.

When tested on RNA, WTX and lynx1 reduce RNA fluorescence only at high concentrations (Figure 8). Similarly to the experiment with DNA, lynx1 produced an effect only at the highest concentration.

**Figure 9:**
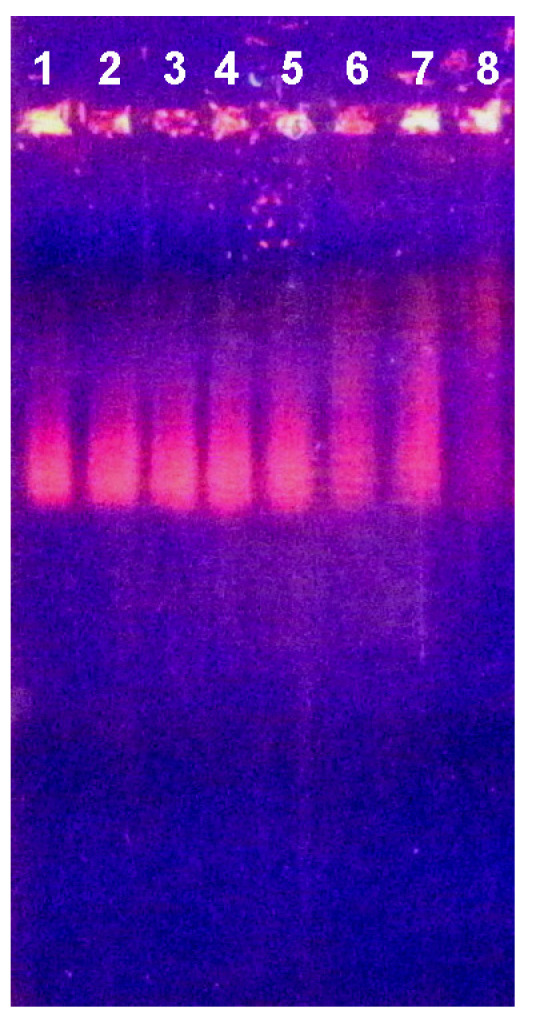
Electrophoretic mobility shift assay (EMSA) of the influence of several toxins on the electrophoretic motility of DNA. Lane 1—control sample of 10 µL DNA (1 mg/mL); lanes 2–8 contain DNA pre-incubated with 1 µL of a 0.5% solution of NTI, lynx1, α-CTX, NTII, WTX, CTI and CTII, respectively.

Based on the EMSA data, one may assume that the capacity of toxins to interact with nucleic acids increases in the order NTI < lynx1 < α-CTX < NTII < WTX < CTI < CTII. This order correlates well with the values of net positive charges of the toxins investigated; pI for NTI is 7.53, for lynx1—8.09, for α-CTX—8.60, for NTII—8.72, for WTX—8.90, for CTI—9.0 and for CTII—9.39. To prove this assumption, an EMSA experiment was performed using equal concentrations of all proteins (Figure 9). As can be seen, the highest effect on DNA mobility was produced by CTII possessing the highest pI, while NTI with the lowest pI was practically inactive.

**Figure 10:**
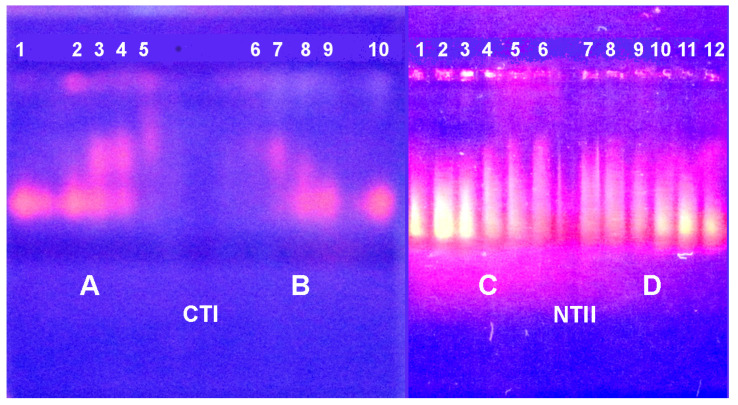
Influence of 2% Tween 20 on the mobility of the DNA–toxin complexes formed after incubation with different concentrations of CTI and NTII. (**A**,**C**)—without Tween 20, (**B**,**D**)—with 2% Tween 20. CTI: lanes 1 and 10—10 µL 1% DNA as control samples; lanes 2 and 9—preincubation with 0.125 µL, lanes 3 and 8—with 0.25 µL, lanes 4 and 7—with 0.5 µL and lanes 5 and 6—with 1 µL of 0.5% CTI. NTII: lanes 1 and 12—control samples; lanes 2 and 11—with 0.5 µL, lanes 3 and 10—with 1 µL, lanes 4 and 9—with 2 µL, lanes 5 and 8—with 4 µL and lanes 6 and 7—with 6 µL of 0.5% NTII.

To test the stability of the toxin–DNA complex, it was incubated in the solution of non-ionic detergent Tween 20. The presence of 2% Tween 20 in the incubation mixtures has little effect on the mobility of the complexes and obviously does not destroy them. The results for the complexes of CTI and NTII with DNA are shown in Figure 10, as examples.

## 3. Discussion

The interaction of DNA or RNA with proteins is a well-known and widespread phenomenon [31]. The best-known examples are histones binding DNA in a nonspecific manner, as well as the proteins involved in the regulation of transcription and translation, binding selectively to strictly defined nucleotide sequences. As discussed in the Introduction, snake venoms contain enzymes affecting nucleic acids which implies direct nucleic acid–protein interaction. On the other hand, natural DNA may affect and inhibit the enzymatic activities of crude snake venom [32,33]. The interaction of DNA with some non-enzymatic proteins from venoms was also documented. Crotamine, a 42-residue basic polypeptide from the venom of the South American rattlesnake *Crotalus durissus terrificus*, was shown to bind DNA in vitro [34,35].

Here, we report that TFTs are able to interact directly with natural nucleic acids. However, it should be noted that the literature already contains indications on the interaction of toxins with nucleotides and synthetic oligonucleotides. A cytotoxin (cardiotoxin 2) from the *N. atra* cobra venom has been shown to bind to ATP and dATP [13], suggesting the possibility of TFT interaction with nucleotide chains rich in adenosine. Indeed, the cytotoxin from the *N. atra* cobra venom binds to Poly-A DNA, leading to the formation of “hairpins” [14]. Interestingly, all five variants of cytotoxins from this venom reproduce the same effect approximately equally, while α-neurotoxins (both “short-chain” and “long-chain”) do not [14], which indicates a specific interaction of cytotoxins with poly-A DNA.

In 2012, for diagnostic purposes, aptamers were developed—synthetic DNA oligonucleotides capable of recognizing α-bungarotoxin, a “long-chain” neurotoxin from krait venom [36]. It was later found that such DNA aptamers recognized variants of cytotoxins from the *N. atra* venom, along with α-bungarotoxin [37]. This example also shows the ability of TFTs to bind DNA.

A long-chain neurotoxin from the Egyptian cobra *N. haje haje* venom induced apoptosis in lymphoblastic leukemia 1301 cells by the mitochondrion-mediated pathway that involves the damage and fragmentation of nuclear DNA [38], but the direct role of the neurotoxin in this process remained unknown. Molecular docking predicts the interaction of a lethal cytotoxin from the venom of *N. kaouthia* (NK-CT1) with the oligonucleotide–human DNA topoisomerase II alpha complex. According to the calculation, amino acid residues Met26, Val27 and Ser28 of NK-CT1 can interact with the nitrogenous bases of the oligonucleotide, which form a complex with DNA topoisomerase Iiα [39].

Based on our results and those published by other groups [13,14,36,37,38,39], we believe that the ability to interact with nucleic acids may be a new general property of TFTs that have a sufficient positive charge on the surface of the molecule. In this work, we show that TFTs form fairly stable complexes with nucleic acids, which were not destroyed either by the addition of a detergent or under ion-pair chromatography conditions.

Obviously, RNA and DNA, which have a total negative charge of the molecule, should form multiple ion bonds with TFTs, which usually have a total positive charge. Indeed, in our experiments, the efficiency of TFTs to bind nucleic acids increased with the increase in their isoelectric point. In general, positively charged amino acids lysine and arginine are located mostly in the central polypeptide loop (finger) of the TFT molecule. In that manner, this part of the TFT molecule may resemble the structure of zinc finger domains, which interact with DNA or RNA in zinc finger proteins known to bind nucleic acids [40,41]. Based on these considerations, one can suggest that animal toxins possessing net positive charge may demonstrate the ability to interact with DNA or RNA. It is quite possible that toxins with positively charged polypeptide loops in their molecular structure may also interact with nucleic acids. Certainly, these assumptions require further experimental proof.

If we consider future directions for research into the interaction of toxins and nucleic acids, then the quantitative characteristics of this interaction should be mentioned. However, this is not a simple task. To quantitatively determine the parameters of the interaction of toxins with nucleic acids, it is necessary to accurately measure the amount of toxin and nucleic acid in the complex, for example, as in the work by Forwood and Jans [42] who used fluorescently labeled compounds. Another direction for further research could be the structural characterization of toxin complexes with DNA/RNA. Currently, various methods are used in the investigation of protein complexes with nucleic acids [43]. The choice of method will depend on the stability of the toxin complex with DNA/RNA. Some pharmacological applications of the detected interaction of TFTs with DNA and RNA can be suggested; for example, TFT may be used to develop a DNA- or RNA-based toxin scavenger. Interestingly, DNA was recently used for the inhibition of *Echis carinatus* venom [33]. At this stage, it is difficult to speak definitely about the biotechnological application of the interaction of TFTs with DNA and RNA. However, one can assume a design of a regulator of the transcription or translation on the basis of the three-finger fold.

Concerning the biological meaning of the found effect, we would like to make the following assumption. TFT molecules have high resistance to proteolysis and can retain functional activity after endocytosis and even after their penetration into both lysosomes and mitochondria [44]. Once TFTs enter the cell, they can interfere with protein biosynthesis by binding to DNA and RNA. Cytotoxins cause cell death through a variety of pathways, including cytolytic action, necrosis, apoptosis and necroptosis [45,46,47]. While the mechanism of cytolytic action is reasonably well understood, the intracellular signaling pathways that lead to cell death are not yet entirely clear. It can be suggested that some stages of these processes involve direct interaction of cytotoxins with nucleic acids. Once in the cell, the cytotoxin targets the mitochondrial membrane (probably by binding to cardiolipin) to promote apoptosis [48]. The neuronal-type nicotinic acetylcholine receptors, some of which are known targets for snake TFT neurotoxins and structurally related lynx1, are expressed in the outer mitochondria membrane to regulate the release of pro-apoptotic substances [49]. In this way, the neurotoxins may enter the mitochondria and disturb their function. It has been shown that exposure to candoxin, a non-conventional TFT neurotoxin from *Bungarus candidus* venom, triggers glial inflammation and neurodegeneration [50]. The molecular mechanism underlying this phenomenon has not been established. Since candoxin caused changes in the expression level of a number of genes [51], it can be hypothesized that it interacts with nucleic acids. So, one can assume that some biological effects of three-finger toxins, which currently do not have a reasonable explanation, may be explained by their interaction with intracellular and probably mitochondrial RNA and/or DNA which follow their internalization after binding to a specific target on the cell membrane.

An interaction of compounds with DNA or RNA can cause various pathophysiological effects, including genotoxicity, genetic damage, chromosomal aberrations, mutagenesis, alterations in DNA replication and damage in the transcription and translation of messenger RNA. It should be noted that these effects have been described at the cellular level only for a few snake venoms and some of their components. Of the TFTs, only cytotoxins have published data that can be correlated with their interaction with DNA or RNA. As previously discussed, cytotoxins induce apoptosis, and more examples can be added. At lower concentrations, cytotoxin sumaCTX from *N. sumatrana* venom triggers apoptosis, independent of its membrane-disrupting effects [52]. It was also found that *N. naja atra* cytotoxins induce ROS generation in SK-N-SH cells, but the cytotoxic potency of CTX3 and CTX4 correlated only in part with their capability to induce ROS generation and mitochondrial alterations [53]. Moreover, cytotoxins can translocate through the plasma membrane and target the outer and inner membranes of mitochondria [54]. While the mechanisms by which cytotoxins can bind to mitochondria are beginning to be explored [54], it is less clear how cytotoxins can cause mitochondrial dysfunction and activate cell death pathways. It is quite possible that at least in part these effects can be explained by cytotoxin interaction with DNA or RNA. Several works describe cell cycle arrest induced by cytotoxins [55,56]. Interestingly, another TFT mambalgin-2 also caused the arrest of the cell cycle in the G1 phase [57]. This effect may also be partly due to the interaction of toxins with DNA or RNA.

With regard to the pathophysiological effects in which nucleic acids are involved, we were unable to find data on the participation of TFTs in these processes, although rich versatile data are available for a number of animal venoms and their components. Thus, toxins of various chemical nature can cause genotoxicity. Bacterial genotoxins have been studied most thoroughly [58], although there are also animal toxins that exhibit genotoxicity. For example, genotoxicity was observed for *Tityus serrulatus* scorpion venom in mice [59]. In human peripheral blood lymphocytes, melittin from bee venom induced cytogenetic damage and changes in gene expression [60]. Toxin Phα1β from the *Phoneutria nigriventer* induced DNA damage in the spinal cord, while its recombinant form increased the micronucleus frequency, which suggests mutagenic effects [61]. Genotoxic effects on human lymphocyte DNA were found for *Bothrops* snake venoms and some toxins isolated from these venoms [62]. No genotoxic effects were reported for Elapidae venoms, which contain TFTs. It is quite possible that TFTs may have genotoxic effects; as cytotoxins penetrate the cell, it is them that can produce these effects. It was shown that a peptide toxin from Russell’s viper *Daboia russelii russelii* venom induced chromosomal aberrations [63]. DNA damage was documented with some animal venoms [64,65] and several isolated toxins [66,67]. Although DNA damage has been found with various venoms and toxins, it may be an indirect effect, including the inhibition or activation of enzymes and proteins involved in nucleic acid metabolism. So far, no direct data demonstrating the participation of TFTs in the considered pathophysiological effects can be found. We hope that our work will stimulate the research in this direction.

## 4. Materials and Methods

### 4.1. Materials

Yeast RNA (fraction IV) was from Merck KGaA (Darmstadt, Germany), high-molecular DNA from cattle spleen was from Biolar (Olaine, Latvia), bovine pancreatic RNase A was from P-L Biochemicals, Inc. (Milwaukee, WI, USA), ethidium bromide was from PanEco (Moscow, Russia), and Tween 20 was from Helicon (Moscow, Russia). All other chemicals obtained from local suppliers were of the highest purity available.

Neurotoxin I (NTI, UniProtKB accession No P01382) [68], neurotoxin II (NTII, UniProt KB No P01427) [69], cytotoxin I (CTI, No P01451) and cytotoxin II (CTII, No P01441) [70] were isolated from the *Naja oxiana* cobra venom and additionally purified by reversed-phase liquid chromatography using Vydac C18 column in the acetonitrile gradient (from 15 to 45% in 30 min) in the presence of 0.1% trifluoroacetic acid. Weak neurotoxin (WTX, No P82935) [71] and α-cobratoxin (α-CTX, No P01391) [72] were isolated from *Naja kouthia* cobra venom. Recombinant human Ly-6/neurotoxin-like protein 1 (lynx1, UniProt KB accession No P0DP58) was obtained as described [73].

### 4.2. Spectrophotometry Measurements

A 1% solution of NTII (or NTI, or WTX, or CTI, or CTII) to final concentrations indicated in Figure 1, Figure 2 and Figure 3 or a 1% solution of bovine pancreatic RNase A to a final concentration of 1 μM was added to 2 mL of a yeast RNA pool solution or high-molecular DNA from cattle spleen at a concentration of 1 ODU/mL in water or a 10 mM glycine buffer, pH 2.7, or Tris-HCl buffer, pH 8.0. A change in optical density was recorded at 260 nm for 10 min using a UV-2450 UV-VIS-spectrophotometer (Shimadzu, Kyoto, Japan). Each measurement was repeated at least three times.

### 4.3. Ion-Paired Reversed-Phase HPLC

A total of 20 µL of 0.6% aqueous RNA solution was incubated overnight with neurotoxin II (50 µg) in the presence of 0.1% detergent Tween 20. The reaction products were analyzed on a Phenomenex C18 Jupiter (4.6 × 250 mm) column (Phenomenex, Torrance, CA, USA) in an acetonitrile gradient from 2 to 22% in 40 min in a 0.1 M triethylammonium acetate buffer, pH 7.0, at a flow rate of 1 mL/min. The chromatography was carried out three times.

### 4.4. Electrophoretic Mobility Shift Assay

Aliquots of 6 µL 0.6% RNA (after centrifugation) and 10 µL 0.1% DNA were incubated for 20 min with different volumes (ranging from 0.125 µL to 6 µL) of 0.5% solutions of CTI, CTII, NTI, NTII, WTX and lynx1. Electrophoresis of the samples with RNA or DNA was carried out in 1% or 0.5% TAE-agarose gel with ethidium bromide at 105 V and 8 W or 6 W in 20 or 15 min, respectively. In a separate set of experiments, 10 µL 0.1% DNA was incubated with the toxins in the presence or absence of 2% Tween20 (final concentration) before electrophoresis. Each experiment was repeated at least three times.

## 5. Conclusions

Using spectrophotometry, ion-paired reversed-phase HPLC and EMSA, we showed that different TFTs from cobra venom possess the ability to interact with DNA and RNA. The efficiency of the interaction correlates with the net positive charge of the toxin molecule. The observed phenomenon may underlie some biological effects of TFTs, which cannot be explained by toxin interaction with their known targets.

## Data Availability

The original contributions presented in this work are included in the article. Further inquiries can be directed to the corresponding author.

## Supplementary Materials

The following supporting information can be downloaded at https://www.mdpi.com/article/10.3390/ijms26094291/s1.

## Abbreviations

The following abbreviations are used in this manuscript:

α-CTX	α-Cobratoxin
CTI	Cytotoxin I
CTII	Cytotoxin II
EMSA	Electrophoretic mobility shift assay
NTI	Neurotoxin I
NTII	Neurotoxin II
TFT	Three-finger toxin
