# Supplementary material for: Cobra Three-Finger Toxins Interact with RNA and DNA: Nucleic Acids as Their Putative Biological Targets
Source: Int J Mol Sci. 2025 May 1;26(9):4291. doi: 10.3390/ijms26094291 (PMC12072136; doi:10.3390/ijms26094291)
Supplement: Supplementary file 1 [file ijms-26-04291-s001.zip › ijms-3542519-supplementary.pdf]

Supplementary materials

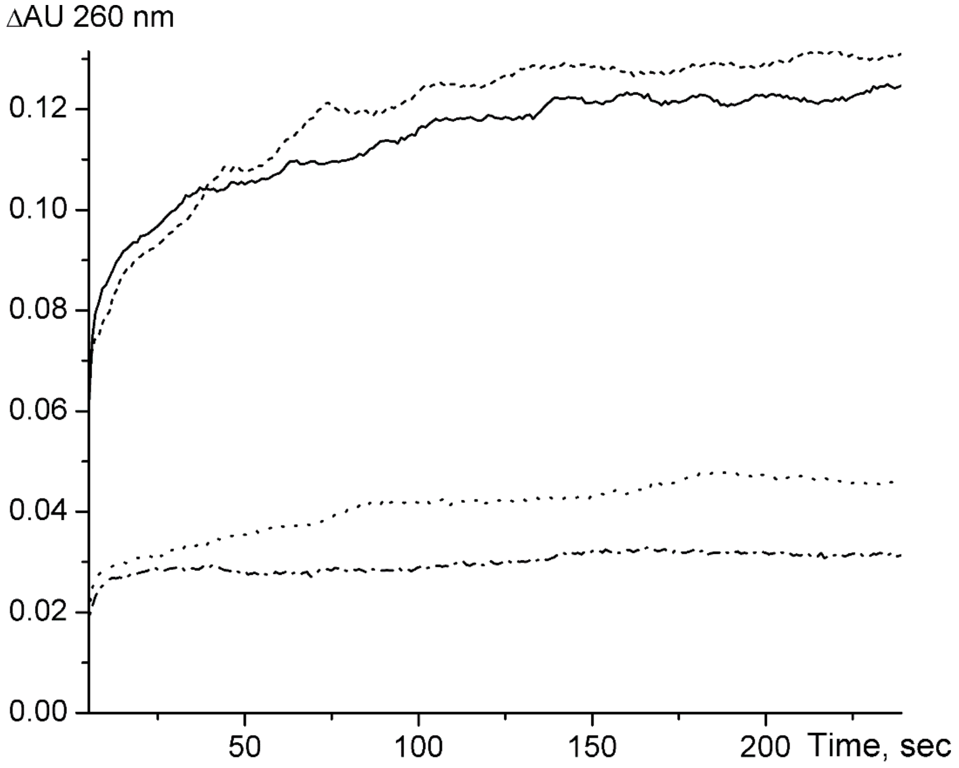

Figure S1. Time-dependent changes in the optical density at 260 nm (ΔmA260) of yeast RNA solution (1 optical density unit) after addition of 1% solution of neurotoxin II (NT II) to a final concentration of 4 μM (dotted and dot-dash lines) and 16 μM (solid and dashed lines). Dashed and dot-dash lines were registered in the presence of 0.1% Tween 20. The background adsorption was subtracted at each curve registration.

Figures S2–S7 in black and white with increased contrast.

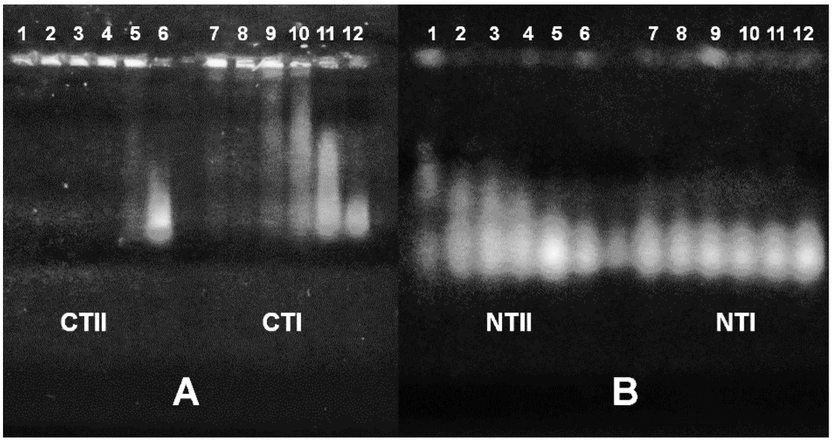

Figure S2. Electrophoretic mobility shift assay (EMSA) of the TFTs influence on electrophoretic motility of DNA. In A, cytotoxins II and I (CTII and CTI) are added in the lanes 1-5 and 7-11, respectively. In B, neurotoxins II and I (NTII and NTI) are added in lanes 1-5 and 7-11, respectively. Control samples (10 µl 0.1% DNA) are in the lanes 6 and 12. The nucleic acid samples were pre-incubated with 4 (lanes 1 and 7), 2 (lanes 2 and 8), 1 (lanes 3 and 9), 0.5 (lanes 4 and 10), and 0.125 (lanes 5 and 11) µl of 0.5% solution of a respective toxin. In this and following figures, the contrast has been increased for better visibility.

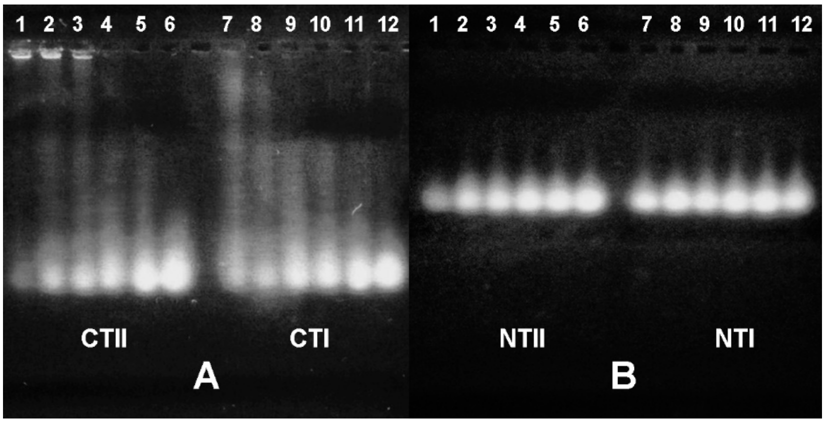

Figure S3. Electrophoretic mobility shift assay (EMSA) of the TFTs influence on electrophoretic motility of RNA. In A, cytotoxins II and I (CTII and CTI) are added in the lanes 1-5 and 7-11, respectively. In B, neurotoxins II and I (NTII and NTI) are added in lanes 1-5 and 7-11, respectively. A control samples (6 µl 0.6% RNA) are in the lanes 6 and 12. The nucleic acid samples were pre-incubated with 4 (lanes 1 and 7), 2 (lanes 2 and 8), 1 (lanes 3 and 9), 0.5 (lanes 4 and 10), and 0.125 (lanes 5 and 11) µl of 0.5% solution of a respective toxin.

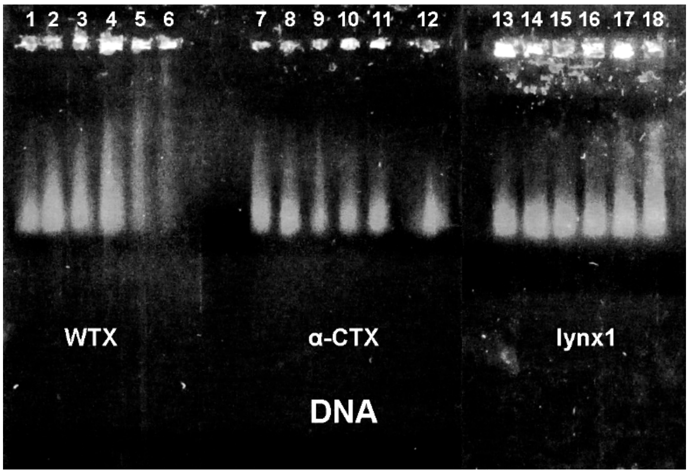

Figure S4. Electrophoretic mobility shift assay (EMSA) of the WTX (left), α-CTX(center) and Lynx1 (right) influence on electrophoretic motility of DNA. The lanes 1, 12 and 13 – control samples of 10 μl DNA (1mg/ml), the left panel); lanes 2, 11 and 14 – DNA with 0. 25 μl, lanes 3, 10 and 15 – with 0. 5 μl, lanes 4, 9 and 16 – with 1 μl, lanes 5, 8 and 17 – with 2 μl, and lanes 6, 7 and 18– with 4 μl of 0.5% WTX, α-CTX or lynx1, respectively.

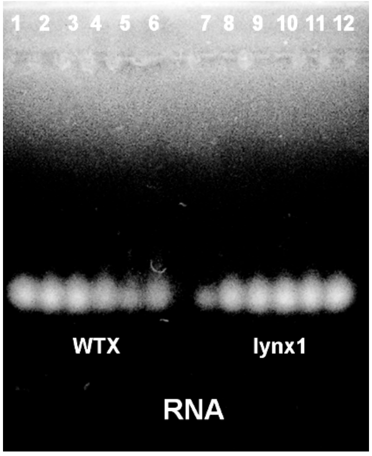

Figure S5. Electrophoretic mobility shift assay (EMSA) of the WTX (left) and lynx1 (right) influence on electrophoretic motility of RNA. The lanes 1 and 12 – control samples of 6 μl RNA (6 mg/ml, the right panel); lanes 2 and 11 – RNA with 0. 25 μl, lanes 3 and 10 – with 0. 5 μl, lanes 4 and 9 – with 1 μl, lanes 5 and 8 – with 2 μl, and lanes 6-7 – with 4 μl of 0.5% WTX or lynx1, respectively.

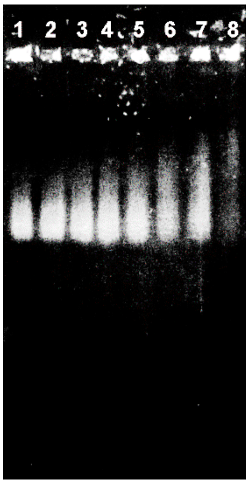

Figure S6. Electrophoretic mobility shift assay (EMSA) of the influence of several toxins on electrophoretic motility of DNA. The lane 1 – control sample of 10 μl DNA (1mg/ml), the lanes 2 – 8 contain DNA preincubated with 1 μl of 0.5% solution of NTI, lynx1, α-CTX, NTII, WTX, CTI andCTII, respectively.

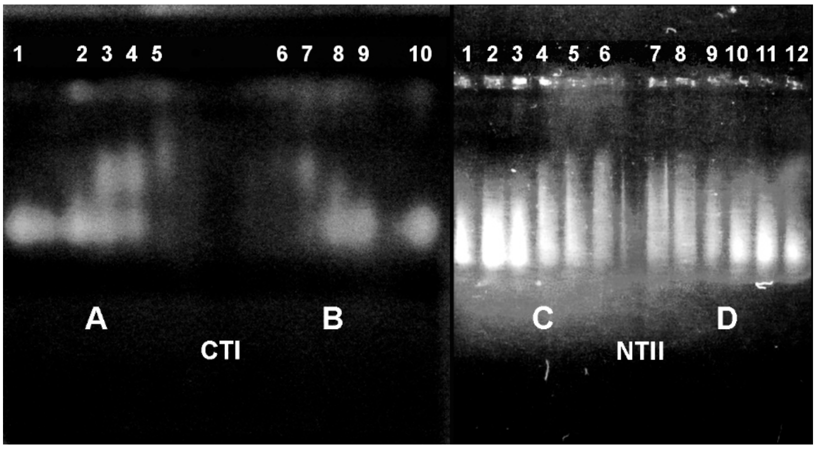

Figure S7. Influence of 2% Tween 20 on mobility of the DNA-toxin complexes formed after incubation with different concentrations of CTI and NTII. A and C – without Tween 20, B and D – with 2% Tween 20. CTI: lanes 1 and 10 – 10 μl 1% DNA as control samples; lanes 2 and 9 – preincubation with 0.125 μl, lanes 3 and 8 – with 0.25 μl, lanes 4 and 7 – with 0.5 μl, and lanes 5 and 6 – with 1 μl of 0.5% CTI. NTII: lanes 1 and 12 – control samples; lanes 2 and 11 – with 0.5 μl, lanes 3 and 10 – with 1 μl, lanes 4 and 9 – with 2 μl, lanes 5 and 8 – with 4 μl, and lanes 6 and 7 – with 6 μl of 0.5% NTII.
